# Long-term outcomes of refractory esophageal strictures after endoscopic submucosal dissection of superficial esophageal neoplasms

**DOI:** 10.1186/s12876-022-02232-x

**Published:** 2022-03-28

**Authors:** Qing Lu, Jin Wang, Xiuhe Lv, Mingjia Xi, Tiantian Lei, Zijing Wang, Li Yang, Jinlin Yang

**Affiliations:** 1 Department of Gastroenterology, West China Hospital of Sichuan University, 37 Guoxue Road, Chengdu, 610041 Sichuan China; 2 Sichuan University-Oxford University Huaxi Gastrointestinal Cancer Centre, West China Hospital of Sichuan University, Chengdu, 610041 Sichuan China

**Keywords:** Endoscopic submucosal dissection, Refractory esophageal structures, Esophageal dilation, Esophageal stents, Long-term outcomes, Prophylactic steroid therapy

## Abstract

**Background:**

Many studies have focused on prophylactic therapy for post-endoscopic submucosal dissection (ESD) of esophageal strictures. However, various strategies cannot prevent the occurrence of postoperative strictures after extensive ESD. Postoperative strictures often inevitably occur, and endoscopic dilation is still a temporarily effective therapy.

**Methods:**

This study included patients with post-ESD refractory esophageal strictures (RESs) from January 2014 to November 2019. Clinical effectiveness was assessed using univariate analysis and multivariate logistic regression. Hierarchical linear models were used to identify factors that predicted the dysphagia-free period.

**Results:**

A total of 50 patients fulfilled the inclusion criteria and entered the study. Twenty-seven (54%) patients had a history of prophylactic oral steroid therapy. Forty-six patients (92%) underwent ≥ 75% circumferential resection, including 32 (64%) cases involving entire circumferential ESD. The mean dysphagia-free period of 50 patients was 2.9 months (95% CI 2.3–3.5). The dysphagia-free period had a linear growth trend over time, increasing by 6.9 days per endoscopic therapy, and the estimated last dysphagia-free period was 85.9 days. Old and female patients had shorter dysphagia-free periods compared with young and male patients. Endoscopic therapy success was achieved in 30 (60%) patients. Multivariate analysis revealed that circumferential lesions (OR 6.106, 95% CI 1.013–36.785, *P* = 0.048) were significant predictive factors for poor clinical outcome.

**Conclusion:**

Endoscopic dilation seemed effective in patients with post-ESD RESs by increasing the dysphagia-free period. After approximately 10 continuous dilations, 60% of patients achieved endoscopic success, and the remission rate of obstruction was increased. Prophylactic oral steroid therapy could reduce the occurrence of RESs. However, once a RES had occurred, prophylactic steroid therapy could not reduce the frequency of dilations or change the long-term outcomes.

*Trial registration:* This study was prospectively registered and approved by the Ethics Committee of West China Hospital of Sichuan University (IRB number: ChiCTR-ONN-17012382) on 2015.

## Background

Endoscopic submucosal dissection (ESD) of superficial esophageal neoplasms, with the advantages of simple performance, minimal invasion, quick recovery, and a high rate of en bloc resection, is used as a substitute for surgery [1]. However, esophageal strictures frequently develop after ESD, especially those involving ≥ 75% circumferential resection [2], which reduces patient survival quality. The occurrence of postoperative strictures for circumferential lesions could approach 100% [3, 4]. In recent years, the majority of researchers have focused on prophylactic therapy for post-ESD strictures, such as oral systemic prednisolone, intralesional steroid injection [5], placement of a fully covered stent after ESD, injection of mitomycin C [6], mesenchymal stem cell culture supernatant [7] or other tissue engineering applications. Although the frequency of strictures was reduced, most strategies could not prevent the occurrence of postoperative strictures after extensive ESD. Postoperative strictures inevitably occurred after large mucosal defects, and patients needed continuing endoscopic dilation for more than two sessions during long-term follow-up [8]. Kochman defined a refractory esophageal strictures (RESs) as more than 3–5 dilations having been performed without clinical and endoscopic responses or when it was impossible to achieve a 14 mm lumen over three dilation sessions [9, 10]. Endoscopic dilation with bougies or balloons was a temporarily effective therapy in patients with RESs [11]. However, the management was time-consuming and challenging, and the patients underwent a long tough period of treatment. Research concerning the quality of life and long-term outcomes of post-ESD RESs is lacking. This allowed us to clearly define the natural history of RESs. Our objective was to explore the long-term outcomes of the patients.

## Materials and methods

### Patients

**Fig. 1:**
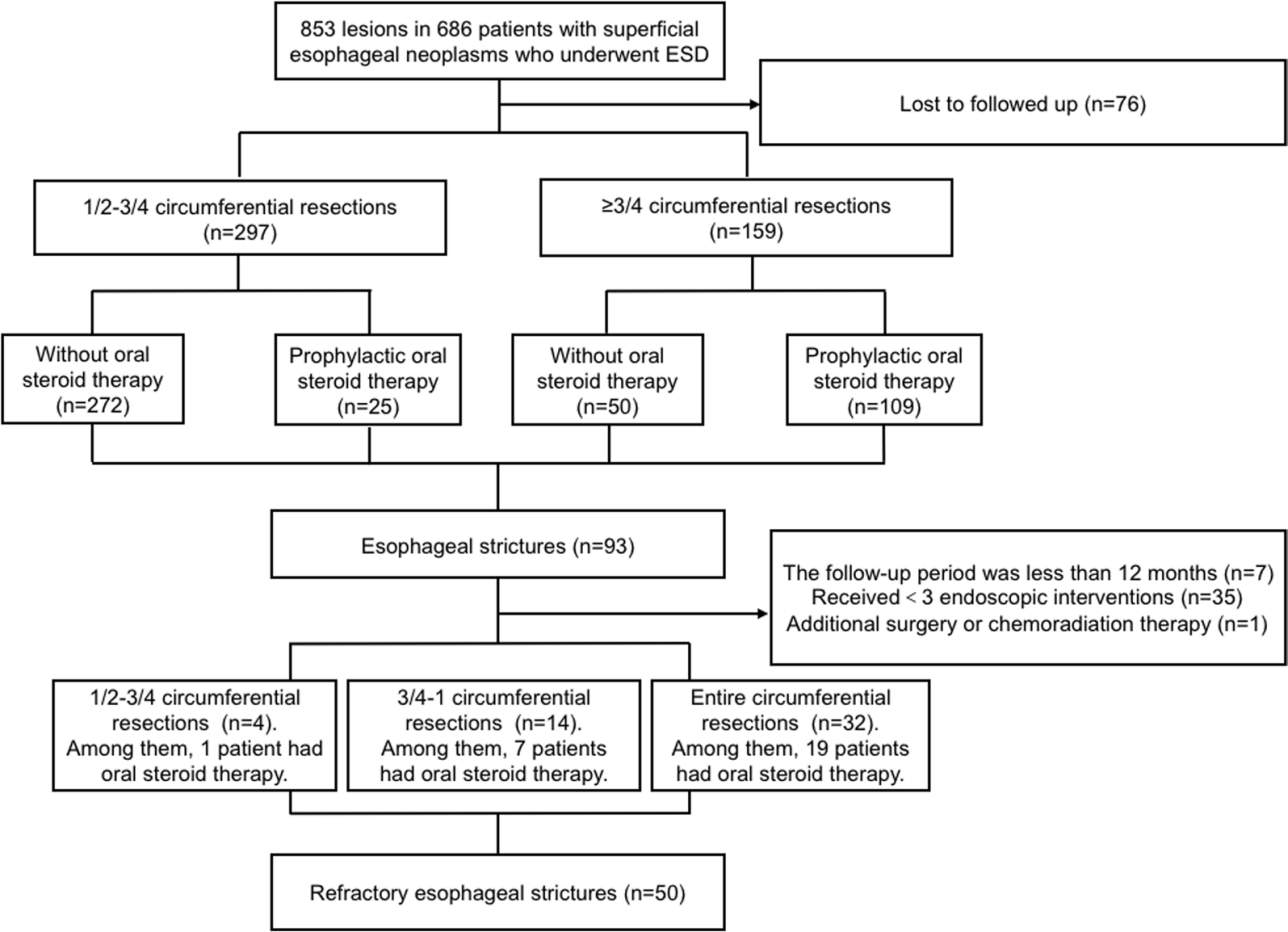
Flowchart of selection of patients with post-ESD refractory esophageal strictures

We prospectively collected the dates of 853 lesions in 686 patients with superficial esophageal neoplasms who underwent ESD in our hospital from January 2014 to November 2019 in our cohort study and retrospectively analyzed it. The inclusion criteria for the study were as follows: (1) pathology indicated low-grade intraepithelial neoplasia (LGIN), high-grade intraepithelial neoplasia (HGIN), or squamous cell carcinoma; (2) no additional surgery or chemoradiation therapy after ESD was applied; (3) no other preventive treatment was given for esophageal strictures except oral steroid therapy; (4) dysphagia occurred after the procedure and esophageal stricture was proven by endoscopy; and (5) symptoms of dysphagia were persistent despite more than or equal to three sessions of endoscopic therapy. The exclusion criteria for the study were as follows: (1) lost to followed up; (2) patients had received < 3 endoscopic interventions; (3) followed-up period was less than 12 months; (4) additional surgery or chemoradiation therapy was applied. Finally, 50 patients were enrolled in the study (Fig. 1). This study was approved by the Ethics Committee of West China Hospital of Sichuan University (IRB number: ChiCTR-ONN-17012382), and informed written consent was obtained from all patients.

### Endoscopic submucosal tunnel dissection procedure

The endoscopic submucosal dissection technique was applied for large superficial esophageal neoplasms with gastroscopes (JIF—Q260 or JIF—Q260J, Olympus, Japan). ESD procedures were performed by experienced endoscopists who had over 10 years of experience in endoscopy. The previous diagnosis of morphology and infiltration depth were inspected by endoscopic ultrasonography (EG-3830 UT, EG-3630 UR, Pantax, Japan) and magnifying endoscopy with narrow band imaging (JIF-H260Z, Olympus, Japan). All patients underwent ESD under general anesthesia with intubation. A dual knife (KD-650 L, Olympus, Japan) marked the tumor margins after iodine staining. The submucosal layer was injected with sodium hyaluronate to lift the lesion. An IT knife (KD-611 L, Olympus, Japan) and a dual knife built the submucosal tunnel from the proximal to the distal side, and en bloc resection was performed though the tunnel. The visible blood vessels were treated by electrocoagulation.

### Management of esophageal stenosis

While patients were fasting, proton pump inhibitors and antibiotics were administered following the ESD procedure. Patients without contraindications used oral steroid therapy to prevent esophageal strictures after extensive ESD. Written consent was obtained before oral steroid therapy. Prednisone was started on postoperative day 2 at a dose of 30 mg/day, with gradual tapering every week (30, 30, 25, 25, 20, 15, 10 and 5 mg/day every week), and then discontinued after 8 weeks [12].

In the post-ESD period, the patients with dysphagia or the inability to achieve a 14 mm lumen underwent dilation procedures with a balloon (5842, 3 ATM-12 mm, 4.5 ATM-13.5 mm, 8 ATM-15 mm, Boston Scientific Corp, America), and salvary bougie dilator (SGD-70–1, length: 70 cm, diameters: 5 mm, 7 mm, 9 mm, 11 mm, 12.8 mm, 14 mm, and 15 mm; Wilson-Cook Medical, America). The dilation session was performed every 2 to 4 weeks until stenosis and dysplasia were alleviated or according to disease recurrence.

A membrane-covered metallic stent (MTN-SE C-membrane; 60–140 m; MicroTech, Nanjing, China) was applied in some cases. The stent was positioned according to the distance from the top of the stenosis to the incisor measured under the endoscope. Stent placement is required to cover 2 cm over both the distal and proximal edge of the narrow section. Stents were removed within 2 months or if adverse events such as perforation occurred.

### Follow-up

Routine endoscopic follow-up was performed at 1, 3, 6, 9, 12 months and then annually after ESD. For patients with strictures, the follow-up was rearranged according to disease recurrence (such as if the gastroscope could not pass through the lumen, dilation would be performed).

### Definitions

The primary outcomes were the clinical resolution of dysphagia and adverse events. Endoscopic therapy success was defined as the maintenance of dysphagia-free status for at least 6 months without any other interventions. Treatment failure was defined as the need for any endoscopic intervention. The secondary outcome was to assess factors that affected the dysphagia-free period. A dysphagia-free period was defined as the time between 2 successive treatments greater than 7 days. The endoscopic interventions within 7 days were considered as only one treatment cycle [13]. Dysphagia was classified into five grades using the Stooler Score as follows [14]: 0, taking a normal diet, no dysphagia; 1, unable to swallow certain solid foods; 2, able to swallow only semisolid soft foods; 3, able to swallow just liquids; 4, unable to swallow liquids in adequate amounts.

### Statistical analysis

We calculated the median or mean and respective ranges for continuous variables. Categorical variables were examined with the chi-square test and Fisher's exact test, and continuous variables were examined with the t test. To estimate the risk factors influencing treatment success, we used logistic regression analysis. The analysis of factors influencing the time trend of the dysphagia-free interval used hierarchical linear models that included both fixed effects and random effects by patients for both intercept and time trends [12]. The temporary interval of treatments for stricture recurrence was used as a substitute for the time variable. The medical data of patients with RESs were divided into two layers. The dilation times were the first layer of data, and the others were the second layer. The model analyzed the effect of time changes for differing levels of other factors. The model was developed as follows: (1) the first layer: Day = β_0_ + β_1_(times) + r and (2) the second layer: β_0i_ = γ_00_ + γ_01_W_1i_ + μ_0i_, β_1i_ = γ_10_ + γ_11_W_1i_ + μ_1i_. Propensity score matching was used to minimize lesion size bias, with 1:1 matching, in the patients with steroid and dilation alone. The matching tolerance was 0.1, and the matching variables were the length of lesion, circumferential ratio, sex and age. The hierarchical linear models used HLM Statistics version 6 (Scientific Software International Inc, Lincolnwood, IL), and all other statistical analyses were performed using SPSS Statistics version 22.0 (IBM Corp, Armonk, NY). *P* < 0.05 was regarded as significant.

## Results

### Subject baseline characteristics

297 patients underwent 50–75% circumferential resection, and 159 patients underwent ≥ 75% circumferential resection. In the patients who underwent ≥ 75% circumferential resection, the percentage of patients with prophylactic oral steroid therapy resulted in RES was 26/109 and without steroid was 20/50 (*P* = 0.041). There were 93 patients (13.6%) who developed strictures by the end time of follow-up. A total of 50 patients fulfilled the inclusion criteria and entered the study. The basic patient characteristics, preoperative pathology, procedure characteristics and treatment received are shown in Table 1. The median (range) duration of the observation period after ESD was 44 (18–77) months. The median age was 65.5 years, and 23 patients were males. In the ESD procedure, the median longitudinal resection length was 9 (2–19) cm, the median operating time was 102 (40–261) min, the rate of en bloc resection reached 100%, the R0 resection rate for these patients was 68%. Muscular injury occurred in 42% of cases, and repeated electric coagulation hemostasis intentionally to prevent posterior bleeding occurred in 48% of cases. Forty-six patients (92%) underwent ≥ 75% circumferential resection, including 32 (64%) cases involving entire circumferential ESD. Regarding postoperative pathology, 20 patients had HGIN, and 30 patients had SCC. All patients were involved the routine endoscopic follow-up. Each biopsy revealed no residual recurrence. Twenty-seven (54%) patients had a history of prophylactic oral steroid therapy. During endoscopic surveillance, the median length of strictures was 4 (1–9) cm. Multiple strictures occurred in 7 cases. The median sessions of dilation were 6 (3–33). The median predilation dysphagia score was 4 (1–4), and the median post dilation dysphagia score was 1 (0–4) (Tables 1, 2).

**Table 1:** The Clinicopathological characteristics of patients with RES after ESD and univariate analysis of endoscopic treatment success

Category		N (%)/M (range)	Success no	P&
Sex	Female	27 (54)	14	0.203
	Male	23 (46)	16	
Age (years)	–	65.5 (45–77)	-	0.921
Smoking history	–	13 (26)	9	0.430
Drinking history	–	12 (24)	7	0.892
Depth of in infiltration	m1	20 (40)	16	0.200
	m2	14 (28)	6	
	m3	12 (24)	6	
	sm1	2 (4)	1	
	sm2	2 (4)	1	
Tumor location*	Cervical	31 (62)	18	0.721
	Thoracic	19 (38)	12	
Postoperative pathology	HGIN	20 (40)	12	0.322
	SCC	30 (60)	18	
Length of lesion (cm)	–	9 (2–19)	-	0.652
Circumferential ratio	1/2–1	18 (36)	13	0.122
	1	32 (64)	17	
Operating time (min)	–	102 (40–261)	–	0.318
En bloc Resection	–	50 (100)	30	-
Muscular injury	–	21 (42)	14	0.413
Clip	–	4 (8)	2	0.670
Coagulation	–	24 (48)	19	0.008
Dilation times	–	6 (3–33)	–	0.763
Length of strictures (cm)	–	4 (1–9)	–	0.643
Number of strictures	One	43 (86)	23	0.020
	Two or more	7 (14)	7	
R0 resection	–	34 (50)	20	0.558
Dysphagia-free period (days)	–	42 (7–863)	–	0.550
Pre-dilation period (days)	–	51 (9–276)	–	0.001
Therapy	Dilation + Oral Prednisone	27 (54)	10	< 0.001
	Dilation only	23 (46)	20	
	Bougie dilator	11 (22)	20	0.022
	Balloon dilator only	39 (78)	10	
	Stents	7 (14)	5	0.506
Pre-dilation dysphagia score	–	4 (1–4)	–	0.098
Post-dilation dysphagia score	–	1 (0–4)	–	< 0.001

RES: Refractory esophageal strictures

^*^The location of oral-lateral lesion

^&^P indicates a significant relationship between characteristics of patients and endoscopic therapy successes in univariate analysis

**Table 2:** The time-trend of dysphagia-free period with post-ESD RES in the model of HLM

Fixed effects		Parameter (SE)	P
Times	Intercept (d)	85.9 (8.4)	< 0.001
	Time-trend (d/time)	6.9 (0.9)	< 0.001
**Patient characteristics**			
*Sex*			
Female–reference category	Intercept (d)	86.1 (8.5)	< 0.001
	Time-trend (d/time)	3.5 (17.2)	0.841
Male	Intercept (d)	7.3 (0.9)	< 0.001
	Time-trend (d/time)	4.5 (1.8)	0.013
*Age*			
	Intercept (d)	86.1 (8.5)	< 0.001
	Time-trend (d/time)	− 1.0 (1.2)	0.416
Slope	Intercept (d)	8.2 (0.9)	< 0.001
	Time-trend (d/time)	− 0.5 (0.2)	< 0.001
**Lesion characteristics**			
*Circumferential ratio*			
≥ 50%, < 100%–reference category	Intercept (d)	86.4 (8.5)	< 0.001
	Time-trend (d/time)	− 12.2 (18.0)	0.500
100%	Intercept (d)	7.1 (1.0)	< 0.001
	Time-trend (d/time)	− 0.75 (2.1)	0.727
*Infiltration depth*			
	Intercept (d)	86.1 (8.5)	< 0.001
	Time-trend (d/time)	− 3.1 (8.2)	0.002
Slope	Intercept (d)	7.2 (0.9)	< 0.001
	Time-trend (d/time)	2.9 (0.9)	0.002
*Stricture length*			
	Intercept (d)	115.8 (19.7)	< 0.001
	Time-trend (d/time)	− 6.4 (3.8)	0.100
Slope	Intercept (d)	9.0 (3.2)	0.005
	Time-trend (d/time)	− 0.3 (0.48)	0.497
*Number*			
One-reference category	Intercept (d)	80.0 (8.0)	< 0.001
	Time-trend (d/time)	39.6 (31.7)	0.099
Two or more	Intercept (d)	7.6 (1.0)	< 0.001
	Time-trend (d/time)	− 2.0 (1.9)	0.292
*Location*			
	Intercept (d)	86.0 (8.5)	< 0.001
	Time-trend (d/time)	7.1 (7.6)	0.689
Slope	Intercept (d)	7.0 (0.9)	< 0.001
	Time-trend (d/time)	0.7 (1.9)	0.730
**Therapy characteristics**			
*Initial dysphagia-free period*			
	Intercept (d)	84.8 (14.9)	< 0.001
	Time-trend (d/time)	0.01 (0.2)	0.924
Slope	Intercept (d)	10.8 (2.1)	< 0.001
	Time-trend (d/time)	− 0.08 (0.04)	0.050
*Therapy*			
Dilation–reference category	Intercept (d)	86.3 (8.3)	< 0.001
	Time-trend (d/time)	− 20.9 (16.8)	0.218
Dilation + Prednisone	Intercept (d)	8.0 (0.9)	< 0.001
	Time-trend (d/time)	− 8.7 (1.8)	< 0.001

RES: Refractory esophageal strictures

SE: Standard error

HLM: Hierarchical linear model

The intercept and time-trend indicate the estimated point and the degree of change

### Clinical outcomes and adverse events

Endoscopic therapy success was achieved in 30 (60%) patients, without the need for further intervention for ≥ 6 months. The average number of dilations in the 30 patients before achieved success was ten dilations. The success rate of patients treated with dilation only (87%, 20/23) was higher than that of patients treated with oral steroid therapy (37%, 10/27). In 20 (40%) patients, the end of follow-up was accompanied by the last dilation. We considered these patients who did not achieve clinical success as defined had poor survival quality, and they were scheduled for subsequent interventions. Five (10%, 5/50) patients experienced food impaction. Four (57%, 4/7) patients experienced stent dysfunction, including stent overgrowth in 1 (14%, 1/7), intolerable chest pain in 1 (14%, 1/7), and stent migration in 3 (43%, 3/7). Two patients died during follow-up, including from suicide and related pulmonary diseases (Table 1).

The results from the univariate analyses suggested that the following factors were associated with success of endoscopic therapy: repeated electric coagulation, number of strictures, predilation period and post dilation dysphagia score. Patients with dilation only were more likely to have clinical resolution than patients with prophylactic steroid therapy, and this difference was significant in the univariate analysis (*P* < 0.001). The multivariate analysis included factors in the univariate analysis and some others meaningful for clinic (Table 3). In multivariate analysis, there was no significant relationship between cervical strictures (OR 0.516, 95% CI 0.121–2.203, *P* = 0.372), depth of infiltration above M2 (OR 1.522, 95% CI 0.372–6.233, *P* = 0.559), muscular injury (OR 0.416, 95% CI 0.091–1.898, *P* = 0.257), or stricture length ≥ 5 cm (OR 0.452, 95% CI 0.084–1.972, *P* = 0.264). Circumferential lesions (OR 6.106, 95% CI 1.013–36.785, *P* = 0.048) were significant predictive factors for clinical resolution (Table 3).

**Table 3:** Multivariate analysis of endoscopic treatment success

Risk factors	OR	95% CI	*P* value
Circumferential ratio (1/2, < 1 vs. 1)	6.106	1.013–36.785	0.048
Location of strictures (Cervical vs. Thoracic)	0.516	0.121–2.203	0.372
Depth of infiltration (m1/m2 vs. m3/sm)	1.522	0.372–6.233	0.559
Muscular injury (no vs. yes)	0.416	0.091–1.898	0.257
Length of strictures (1–4 cm vs. 5–9 cm)	0.452	0.084–1.972	0.264

### Dysphagia-free period

**Fig. 2:**
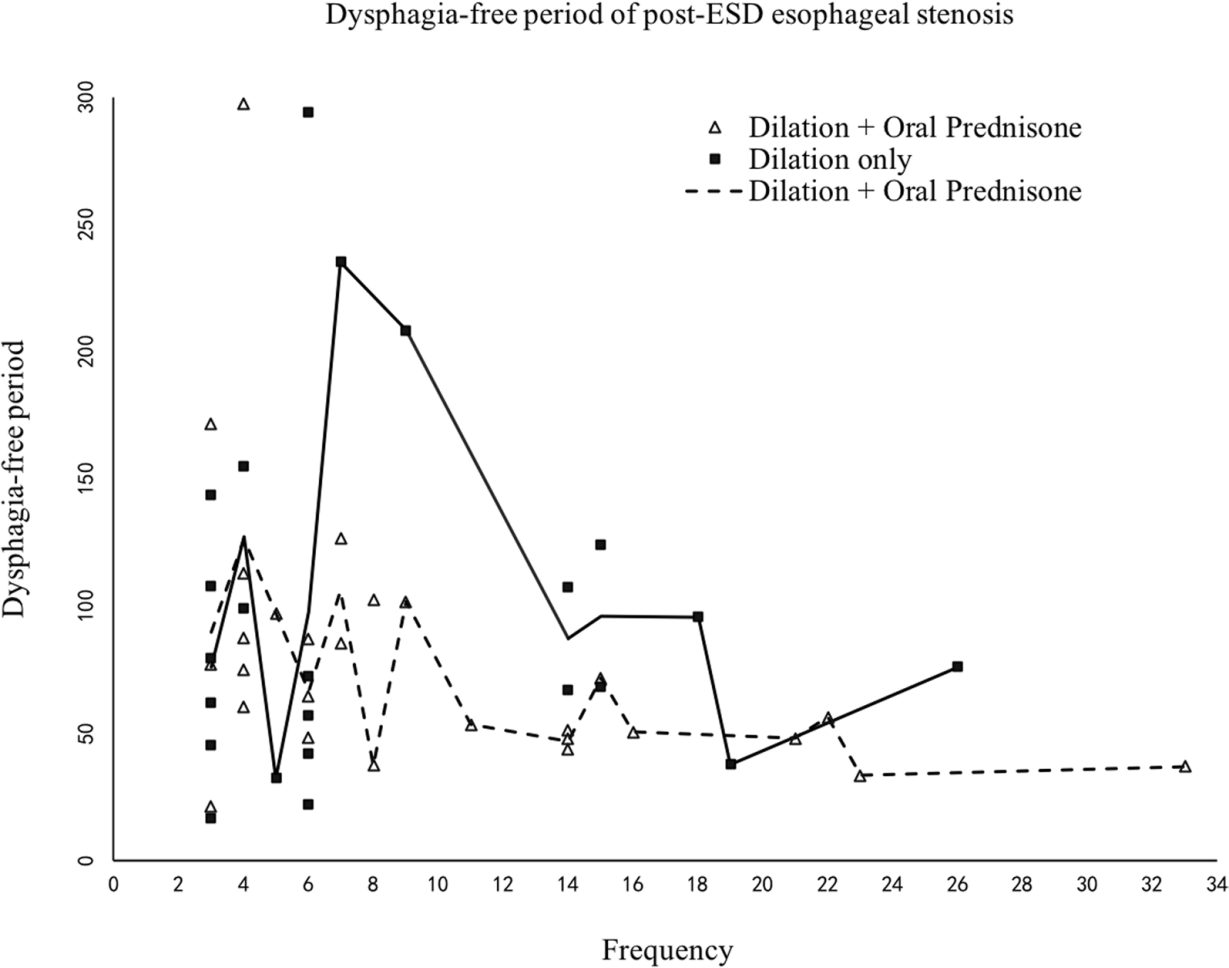
Dysphagia-free period of post-ESD esophageal stenosis

The mean dysphagia-free period of the 50 patients was 2.9 months (95% CI 2.3–3.5). The dysphagia-free period of patients with oral steroid therapy was 2.7 months (95% CI 1.9–3.4), and the dysphagia-free period of patients with dilation only was 3.3 months (95% CI 2.2–4.2). There was no significant relationship between dysphagia-free period of patients with and without oral steroid therapy (*P* = 0.304). The mean dysphagia-free period after dilation only was higher than that with oral prednisone. The less dysphagia-free period the patients experience, the more likely multiple dilation procedures will be required (Fig. 2). Propensity score matching was used to minimize lesion size bias. Finally, the 36 patients were matched, with 18 patients in each group. After adjusting influence on the two groups, the dysphasia-free period of steroid group was 2.8 months, and the dilation alone group was 3.1 months (*P* = 0.129).

To explore a trend in the dysphagia-free period over time, we used hierarchical linear models (Table 2). The result of the fixed effect intercept and slope was significant. The mean dysphagia-free period for the entire sample was not constant over time, but there was a linear growth trend over time, increasing by 6.9 days per endoscopic therapy. The estimated last dysphagia-free period was 85.9 days. We included patient, lesion and therapy characteristics in the second level to estimate independent prognostic factors associated with the trend in the dysphagia-free period. There was no significant relationship between the dysphagia-free period time trend and the circumferential ratio, lesion location, stricture length, stricture number or initial dysphagia-free period. However, sex (4.5, *P* = 0.013), age (− 0.5, *P* < 0.001), infiltration depth (2.9, *P* = 0.002) and therapy (− 8.7, *P* < 0.001) tended to predict the slope of the dysphagia-free period time trend. Old and female patients had shorter dysphagia-free periods compared with young and male patients. The patients who received oral steroid therapy had shorter dysphagia-free periods than those who received dilation therapy alone. However, for infiltration depth, the intercept and slope displayed contrary tendencies. Therefore, the infiltration depth should not simply predict the trend (Table 2).

## Discussion

In a previous study, Kochman defined a RES as more than 3–5 dilations having been performed without clinical and endoscopic response [15]. In fact, the exact number of additional dilation sessions required to categorize the RESs lacked a unified standard in the references [16–18]. Most patients with 3 dilation sessions still required constant endoscopic treatment after the follow-up in our study. Therefore, we restricted the definition of a RES to at least 3 endoscopic treatment sessions.

We found that endoscopic therapy tended to be effective in patients with RESs by increasing the dysphagia-free period per dilation. In previous studies, the dysphagia score was often used as the endpoint, without a consistent standard. However, the mean length of the dysphagia-free period was the proxy for survival quality in our study. We found that the dysphagia-free period had a linear growth time trend, increasing by 6.9 days per endoscopic therapy. Factors including elderly age and female gender were associated with shorter dysphagia-free period. The definite reasons for this were unclear and awaiting further study.

In our study, the patients who received oral steroid therapy had shorter dysphagia-free periods than those who received dilation therapy alone. We analyzed the baseline data and did not find significant differences in two groups, except the lesion size. The dysphasia-free period may be influenced by this. Propensity score matching was used to minimize lesion size bias. After adjusting influence on the two groups, we found the dysphasia-free period of steroid group was 2.8 months, and the dilation alone group was 3.1 months. There was no statistical difference between the two. Therefore, the steroid group still had longer period. After considering this effect, we assume that there is probably the same dysphasia-free period between the two group with larger sample size. However, our study still suggest that prophylactic steroid therapy could not reduce the frequency of dilations or change the long-term outcomes.

At present, prophylactic oral steroid has become a common clinical treatment to prevent esophageal stricture after ESD, because of inhibiting inflammation response, collagen synthesis and fibroblast proliferation [19]. Yamaguchi et al. conducted many clinical trials, which proved for the first time that oral steroids have effect on esophageal stricture after ESD [12]. The therapy is more economical, effective and less painful comparing repeated dilations. But there is not 100% safe. Severe complications could occur, for example, pulmonary infection, immunosuppression, elevated blood sugar, osteoporosis. In our study, prophylactic oral steroid therapy had lower occurrence of RESs. This was proved by previous literatures. As for steroid injections, we had no similar experience, but previous literatures suggested that steroid injections were useful and had some high-risk complications [20].

In the long-term follow-up, three-fifths of the patients achieved endoscopic therapy success, and two-fifths of the patients required constant endoscopic treatment before the end of the follow-up. Literature focused on the long-term outcomes of RESs are scarce. This allowed us to clearly define the final end of RESs. In our study, endoscopic treatment success demonstrated better survival quality and a schedule for subsequent interventions at least 6 months later. We found that three-fifths of the patients could achieve a better quality of life, and as much as one-year dysphagia-free time was reached in some patients. However, two-fifths of the patients remained dependent on repeated dilation sessions. The univariate analysis revealed that prophylactic steroid therapy could not change the long-term outcomes of RESs, and most patients did not achieve endoscopic success. It was different from past cognition. Many studies have shown that steroid therapy could decrease the incidence of post‐ESD strictures in high‐risk patients [21]. However, we found that prophylactic steroid therapy could not play a role in the long-term natural history of RESs once strictures occurred. In fact, the rate of successful outcome appeared to be worse in the subgroup with prophylactic steroid therapy than in the subgroup without prophylactic steroid therapy.

Another finding was that the apparently promising alternative to dilation endoscopic stenting does not affect the long-term natural history of the disease. In the multivariate analysis, we found that circumferential lesions were a predictor of endoscopic treatment failure. In a previous study, the circumferential resection range was identified as an independent risk factor for post-ESD esophageal strictures [12]. Previous literature suggests that the incidence of strictures is between 80 and 100% in patients with a circumferential mucosal defect > 75% [22]. A tumor circumference > 75% was an independent risk factor for refractory strictures [23]. The occurrence of strictures after ESD for circumferential lesions could approach 100%. Our results demonstrated that the circumferential ratio could also predict the clinical resolution of RESs. The probability of endoscopic treatment failure was high in patients with entire circumferential ESD who had poor survival quality and needed dilation in a short time.

Although endoscopic therapy tended to be effective by increasing the dysphagia-free period per dilation, nearly all patients with RESs after ESD experienced a long tough period. In our study, the maximum sessions of dilation were 33. After nearly five years of suffering, the dysphagia-free time of the patient with 33 sessions increased from one month to more than one year, which we considered achieving clinical success as defined in this study. However, most patients needed several sessions of dilations in a short period, resulting in anxiety and depression. One patient with 8 sessions lost confidence and chose to commit suicide.

We included patients from January 2014 to November 2019. In fact, dilation alone group was collected before 2017, and oral steroid group was collected after 2017. The main reason for this is that many studies around 2017 demonstrated the prophylactic effect of oral steroid on stenosis formation. Since 2017, patients with large-area resection needed oral steroid therapy. But there were no statistically significant differences in baseline data between patients with and without oral steroid.

This study has several limitations. First, our study had a single-center design. We collected data prospectively and analyzed it retrospectively. Second, we only discussed the efficacy of balloon dilation, bougie dilation, stent and prophylactic oral steroid, without involving steroid injection and mitomycin C. Third, in multivariate analysis, 5 explanatory variables might be a bit overfitting for 30 cases with endoscopic therapy successes. A large, multicenter study is needed to identify predictors of clinical results and dysphagia-free periods in the future.

## Conclusion

Endoscopic dilation seemed effective in patients with post-ESD RESs by increasing the dysphagia-free period. After approximately 10 continuous dilations, 60% of patients achieved endoscopic success, and the remission rate of obstruction was increased. However, once a RES had occurred, prophylactic steroid therapy could not reduce the frequency of dilations or change the long-term outcomes.

## Data Availability

The datasets used and/or analyzed during the current study are available from the corresponding author on reasonable request.

## Abbreviations

ESD: Endoscopic submucosal dissection
RESs: Refractory esophageal strictures
LGIN: Low-grade intraepithelial neoplasia
HGIN: High-grade intraepithelial neoplasia
